# Harnessing Antioxidants in Cancer Therapy: Opportunities, Challenges, and Future Directions

**DOI:** 10.3390/antiox14060674

**Published:** 2025-05-31

**Authors:** Yu’e Liu, Guangzhen Wu, Linjing Feng, Jialing Li, Yuyang Xia, Wenjia Guo, Kaijun Zhao

**Affiliations:** 1 Department of Neurosurgery, Shanghai East Hospital, School of Medicine, Tongji University, Shanghai 200120, China; 2 Boston Children’s Hospital, Dana Farber Cancer Institute, Harvard Medical School, Boston, MA 02115, USA; 3 Shanghai Fourth People’s Hospital, School of Medicine, Tongji University, Shanghai 200434, China; wuguangzhen328@163.com; 4 Department of Oncology Surgery, Shanghai Mengchao Cancer Hospital, Shanghai University, Shanghai 201800, China; fenglinjing1125@163.com; 5 University of Illinois College of Medicine Rockford Family Medicine, Rockford, IL 61104, USA; li198@uic.edu; 6 School of Medicine, I.M. Sechenov First Moscow State Medical University, 119991 Moscow, Russia; yuyangx02@gmail.com; 7 Department of Laboratory Medicine, Shanghai East Hospital, School of Medicine, Tongji University, Shanghai 200092, China; 13002219875@163.com

**Keywords:** antioxidant, Bach1, NRF2, ROS, cancer therapy

## Abstract

Antioxidants neutralize reactive oxygen species and free radicals, protecting cells from oxidative damage and maintaining cellular homeostasis. In cancer therapy, they play a complex dual role, serving as protective agents against oxidative stress while, under certain conditions, acting as pro-oxidants that may promote tumorigenesis and resistance to treatment. Redox regulation is governed by key antioxidant pathways, such as the BACH1 and NRF2 pathways, along with transcriptional factors that significantly affect cancer progression and immunotherapy response. In addition to their intracellular effects, antioxidants modulate the tumor microenvironment, including interactions with the extracellular matrix, which impact cancer cell behavior and therapeutic responses. This review also explores preclinical studies that investigate the roles of major antioxidants in cancer biology. While these studies present promising data, significant challenges persist, including the potential for antioxidants to interfere with standard cancer treatments or to inadvertently support tumor survival. We further highlight emerging strategies aimed at optimizing antioxidant therapy, including personalized medicine approaches, nanoparticle-based delivery systems, and combination treatments with immunotherapies and targeted therapies. By examining the therapeutic potential and associated risks of antioxidants, this review provides critical insights into their role in cancer treatment and offers a roadmap for advancing antioxidant-based strategies to improve clinical outcomes.

## 1. Introduction

Various strategies for the treatment of cancer have been developed; besides traditional surgery, chemotherapy, and radiotherapy, novel immunotherapy and targeted therapies have improved survival rates for many cancer patients. However, these therapies are often limited by toxicity, tumor resistance, and tumor recurrence, emphasizing the need for more refined, effective, and personalized treatments. The complexity of the tumor microenvironment (TME) has become increasingly apparent, highlighting the necessity for therapeutic strategies that not only target the cancer cells directly but also modulate the TME. Among all the elements in the TME, the redox balance plays a fundamental role in orchestrating cellular processes such as growth, survival, and drug resistance [1]. Tumor cells often exhibit elevated levels of reactive oxygen species (ROS), which they use to drive proliferation and metastasis, but excessive ROS also induce cell death. Therefore, targeting the redox balance provides a double-edged approach—either by increasing ROS to toxic levels or by impairing antioxidant defenses. For example, drugs such assuch as Buthionine Sulfoximine (BSO) deplete glutathione, a major intracellular antioxidant, thereby sensitizing tumor cells to oxidative stress and enhancing the efficacy of chemotherapy [2,3]. Targeting the redox balance within tumors may offer new opportunities to enhance treatment efficacy and overcome current therapeutic limitations [4,5].

ROS have emerged as critical modulators of cancer progression and response to therapy [6,7]. ROS are metabolic byproducts generated during cellular energy production processes, primarily within the mitochondria through oxidative phosphorylation [8,9]. ROS account for approximately 1–2% of the overall oxygen consumption in healthy cells [10]. In contrast, cancer cells typically show significantly elevated levels of ROS compared to normal ones attributed to mitochondrial dysfunction, oncogenes activation, and an imbalance in antioxidant defense mechanisms. These highly reactive molecules, which include free radicals and non-radical species such as hydrogen peroxide, are involved in numerous cellular processes, ranging from cell proliferation and DNA damage to apoptosis and senescence [11,12]. Importantly, ROS play a dual role in cancer development, serving as both facilitators and inhibitors of oncogenesis. While low levels of ROS promote tumorigenesis via activating oncogenes and inactivating tumor suppressors, high ROS trigger oxidative stress, resulting in cellular damage, DNA mutations, and cancer cell ferroptosis [13,14]. Meanwhile, high ROS levels promote the suppressive function of myeloid-derived suppressor cell (MDSC) on the immune response; they not only facilitate MDSC-induced immune suppression but also initiate the differentiation and polarization of M2-type tumor-associated microphages [15]. Thus, ROS are drivers of cancer initiation and progression, as well as effectors of cancer therapy, contributing to the efficacy of treatments such as chemotherapy and radiotherapy.

Given this paradoxical role, antioxidants have been regarded as a potential therapeutic strategy in oncology [16]. The role of antioxidants in cancer therapy is complex. Recent research has moved beyond the simple classification of antioxidants as either “beneficial” or “harmful” to recognize their context-dependent actions, which vary based on factors such as tumor type, treatment protocols, and the tumor microenvironment [17]. In this review, we discuss several critical pathways that control the antioxidant level in cells, and the dual role of antioxidants in cancer therapy. Then, we explore strategies for using antioxidants in cancer therapies. We discuss recent advancements in targeted antioxidant therapies, nanoparticle-based delivery systems, and combination treatments with emerging therapies.

This review primarily focuses on preclinical studies of antioxidants in cancer therapy and discusses their translational relevance to clinical applications. It highlights how the precision application of antioxidants, informed by personalized medicine and biomarker identification, may improve cancer treatment outcomes while minimizing associated risks.

## 2. The Regulation of Antioxidant Pathways

The regulation of antioxidant pathways is central to the dynamic balance between oxidative stress and cellular redox homeostasis in cancer cells. Antioxidants play a pivotal role in counteracting the damaging effects of ROS, which are inherently linked to tumorigenesis, cancer progression, and the response to cancer therapies [6]. Cancer cells often adapt their antioxidant systems not only to cope with the increased ROS levels associated with rapid proliferation but also to evade the cytotoxic effects of therapies designed to generate oxidative stress [18]. This section summarizes key transcription factors involved in antioxidant regulation, including the Bric-à-brac domain and CNC homolog 1 (BACH1), nuclear factor erythroid 2-related factor 2 (NRF2), hypoxia-inducible factor (HIF), and p53 pathways, while also highlighting the role of the PI3K-Akt pathway in managing oxidative stress in cancer cells.

### 2.1. BACH1 Suppresses the Antioxidant Genes Expression

To cope with the free radicals and ROS, cells have evolved a defense system to maintain redox balance through antioxidant responses. BACH1 and NRF2 are two of the most important genes known so far for determining the antioxidant levels in cells. BACH1 serves as a strong repressor of antioxidant genes, while NRF2 acts an activator under oxidative stress. Together, they compete for binding to antioxidant response elements (AREs) to modulate the expression of downstream genes.

As a redox-sensitive transcription factor, BACH1 plays a crucial role in regulating genes involved in angiogenesis [19]. Under normal conditions, BACH1 predominates and maintains a resting state by suppressing genes associated with redox balance; it binds to AREs and suppresses downstream antioxidant genes expression. However, when oxidative stress intensifies, NRF2 is activated and translocates to the nucleus, where it triggers the rapid expression of antioxidant genes such as heme oxygenase-1 (HO-1), which plays a central role in heme degradation. Simultaneously, the oxidation of heme-containing proteins results in the release of free heme. When heme accumulates in the cell, it binds to BACH1, prompting BACH1 to translocate from the nucleus to the cytoplasm; then, BACH1 is quickly degraded through the ubiquitin-proteasome pathway [20]. This process is dually regulated by complementary SCF-type E3 ligase and finally results in the loss of BACH1’s transcriptional activity, as it no longer binds to AREs, thereby reducing its inhibitory effect on related genes [21]. Under mild oxidative stress, BACH1 is stabilized by low levels of ROS and low levels of heme. It displaces NRF2 from AREs and acts as a transcriptional suppressor of antioxidant genes [22]. This complex feedback loop not only safeguards cells against oxidative stress damage but also prevents excessive activation of the antioxidant mechanisms.

**Figure 1:**
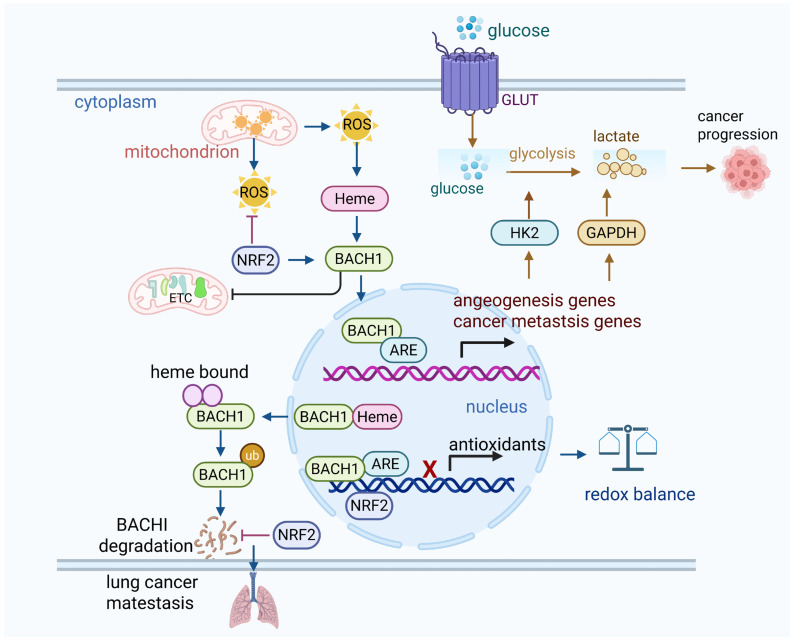
BACH1 suppresses the antioxidants. (1) Under normal oxidative status, BACH1 keeps the oxidative balance by binding with antioxidant response elements in nucleus. (2) Under oxidative stress, ROS stabilize BACH1, while BACH1 competes with NRF2 to bind the AREs in the nucleus and suppress the antioxidant genes. High oxidative stress actives NRF2, and NRF2 binds to AREs and activates downstream antioxidative enzymes such as HO-1. Thus, BACH1 is translocated from the nucleus to cytosol due to its binding to heme, and it is subsequently degraded by the proteasome. This further reduces BACH1’s ability to inhibit antioxidant gene expression. (3) Activation of BACH1 upregulates cancer cell glycolysis and suppresses mitochondrial oxidative phosphorylation, thus leading to lung cancer metastasis. Similarly, depletion of BACH1 sensitizes breast cancer cells to mitochondria oxidative phosphorylation inhibitors. NRF2 activation enhances lung cancer metastasis by preventing the degradation of BACH1.

The antioxidant treatment eliminates low levels of ROS and enhances angiogenesis gene expression in cancer cells, xenograft tumors, and tumor organoids in a BACH1-dependent manner under normoxic conditions [23]. BACH1 levels increase under hypoxic conditions and with prolyl hydroxylase inhibitors, even in HIF1α-knockout cells, although the role of BACH1 in promoting angiogenesis is independent of HIF1α [23]. High level of BACH1 makes tumors more sensitive to anti-angiogenesis therapies in lung cancer [23]. Moreover, antioxidants promote cancer metastasis by lowering the free heme levels and stabilizing BACH1. BACH1 subsequently enhances the expression of Hexokinase 2 (HK2) and Gapdh, leading to increased glucose uptake, accelerated glycolysis, and elevated lactate production. This metabolic reprogramming drives glycolysis-dependent metastasis in lung cancer cells [24]. Similarly, in breast cancer, BACH1 reduces glucose metabolism in the tricarboxylic acid (TCA) cycle and represses the transcription of genes involved in the electron transport chain (ETC). Knockdown of BACH1 by shRNA or its degradation by hemin enhances the sensitivity of cells to ETC inhibitors such as metformin [25,26]. Thus, targeting BACH1 restored glycolysis and inhibited metastasis induced by antioxidants (Figure 1).

### 2.2. NRF2 Pathway Activates Antioxidant Gene Expression

**Figure 2:**
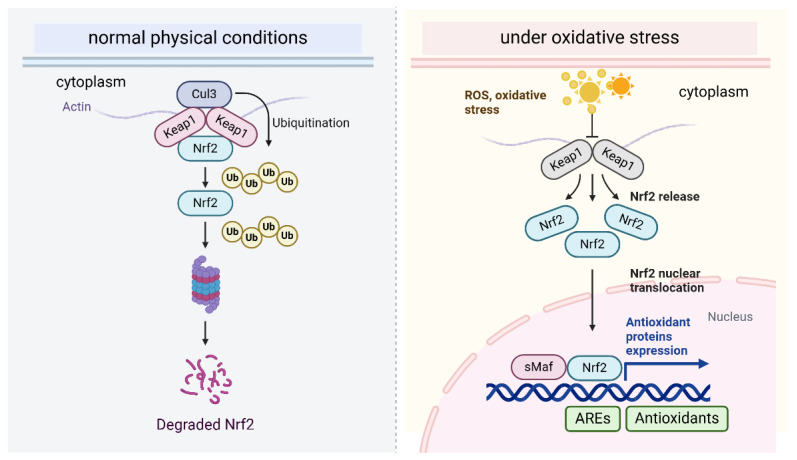
The Nrf2-Keap1 pathway regulates oxidative stress.

Nrf2 is an essential transcription factor involved in the cellular defense mechanisms against oxidative stress, primarily by regulating the expression of antioxidant response genes. Under basal physiological conditions, Nrf2 levels are kept low through its association with Kelch, such as ECH-associated protein 1 (Keap1), which acts as an adaptor in the Cul3 (Cullin 3)-dependent ubiquitin E3 ligase complex, facilitating its degradation [27]. Keap1 functions as a negative regulator by targeting Nrf2 for proteasomal degradation, thereby preventing its accumulation in the cell. This regulatory mechanism ensures that NRF2 activity remains low under basal conditions, preventing unnecessary antioxidant activation. Keap1 contains multiple cysteine residues, including Cys151, Cys273, and Cys288, which are sensitive to oxidative stress [28]. Upon the accumulation of ROS, these cysteine residues undergo oxidation, leading to a conformational change in Keap1 [29]. This modification interferes with the interaction between Keap1 and Nrf2, a process mainly mediated by the Kelch domain of Keap1 and the Neh2 domain of Nrf2. The oxidation of the cysteine residues in Keap1 reduces its binding affinity for the ETGE and DLG motifs in the Neh2 domain of Nrf2. This weakening of the interaction facilitates the dissociation of Nrf2 from Keap1, leading to its stabilization. Once released from Keap1, Nrf2 translocates to the nucleus, where it forms a heterodimer with small Maf proteins and subsequently binds to ARE in the promoters of various genes involved in cellular protection [30,31]. The ARE sequences, located in the promoters of genes that encode critical antioxidant enzymes such as glutathione S-transferase (GST), NAD(P)H quinone dehydrogenase 1 (NQO1) [32], superoxide dismutase (SOD) [32], and catalase, play a crucial role in the cellular response to oxidative stress. When Nrf2 binds to ARE, it activates the transcription of genes that collectively aid in ROS detoxification and reduce oxidative damage to cellular components, thereby supporting cell survival under oxidative stress conditions. Moreover, Nrf2 also regulates the expression of other crucial molecules involved in redox homeostasis, including glutathione, thioredoxins, and various detoxifying enzymes (Figure 2).

In normal tissues, this NRF2-driven antioxidant response serves a cytoprotective function by preventing oxidative damage, preserving genomic stability, and maintaining cellular homeostasis. However, in cancer cells, the role of NRF2 becomes contextually and temporally distinct. Many tumors exhibit constitutive activation of NRF2, which aids tumor cells in surviving the elevated oxidative stress associated with rapid proliferation and inflammation [33]. By enhancing antioxidant gene expression, Nrf2 helps tumor cells maintain redox balance and resist cell death induced by ROS, which is crucial for tumor survival, growth, and resistance to chemo- and radiotherapies [34]. For instance, the activation of Nrf2 potentiates lung cancer metastasis by inhibiting the heme- and Fbxo22-mediated degradation of Bach1 [35]. Moreover, Nrf2 activation contributes to cancer progression by facilitating tumor cells’ invasion into surrounding tissues and remodeling the tumor microenvironment to support continued growth [36,37]. The activation of Nrf2 facilitates the polarization of tumor-associated macrophages (TAMs) toward the M2 phenotype to support tumor metastasis [38]. However, under asparagine restriction, Nrf2 activation in CD8^+^ T cells confers a robust growth of T cells and potentiates the T cell-mediated anti-tumoral response [39]. Additionally, Nrf2 activation helps cancer stem cells evade chemotherapy-induced apoptosis [40].

Inhibiting Nrf2 sensitizes tumor cells to ROS-dependent treatments, such as chemotherapy and radiation [41]. Several strategies are being explored to inhibit Nrf2, including disrupting its interaction with Keap1 or preventing its nuclear translocation. Conversely, activating Nrf2 may offer protective benefits to normal cells during treatment, potentially reducing side effects. For example, the natural antioxidant quercetin binds with KEAP1 at Arg415 and Arg483 to inhibit macrophage pyroptosis by activating NRF2 [42]. In summary, NRF2 serves as a double-edged sword in redox regulation: while it protects normal cells from oxidative damage, its sustained activation in cancer cells promotes tumor progression and therapy resistance. Understanding the spatial and temporal dynamics of NRF2 activation is therefore essential for designing selective redox-targeted cancer therapies.

This figure depicts the NRF2-KEAP1 signaling pathway, which governs the cellular antioxidant response. Under basal conditions, NRF2 is sequestered by KEAP1, leading to its degradation. Upon oxidative stress, reactive oxygen species (ROS) modify KEAP1, triggering a conformational shift that releases NRF2, allowing it to stabilize. NRF2 then translocates to the nucleus, where it associates with antioxidant response elements (ARE) and activates the transcription of genes that encode cytoprotective proteins, such as antioxidant enzymes, to mitigate oxidative damage.

### 2.3. Hypoxia-Inducible Factor (HIF) Pathway

#### 2.3.1. Working Mechanism of HIF Pathway

The HIF pathway intersects with the antioxidant response to maintain cellular redox balance and safeguard cells against oxidative damage [43,44]. Under normoxic conditions, prolyl hydroxylase domain (PHD) enzymes facilitate the hydroxylation of HIF-α subunits, such as HIF-1α. This modification allows the von Hippel–Lindau (VHL) protein to recognize HIF-α, promoting its ubiquitination and subsequent degradation via the proteasome [35]. However, under hypoxic conditions, the inhibition of PHD enzymes prevents hydroxylation of HIF-α, allowing it to accumulate and remain stable. Once stabilized, HIF-α translocates to the nucleus, where it forms a dimer with the HIF-β subunit. This complex binds to hypoxia-response elements (HREs) in the promoter regions of target genes, activating the transcription of genes associated with hypoxic adaptation, metabolic regulation, and oxidative stress response [45].

#### 2.3.2. HIF Cooperates with NRF2 to Deal with Oxidative Stress Under Chronic Hypoxia

**Figure 3:**
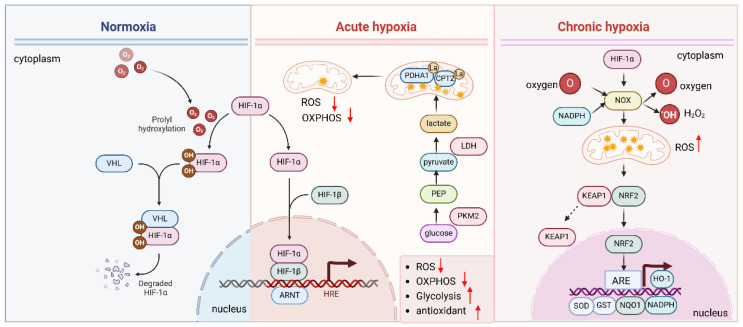
Hypoxia and HIF regulate antioxidants.

HIF plays a crucial role in regulating antioxidant gene expression in response to hypoxia. Chronic hypoxia induces oxidative stress by increasing ROS production. To counteract ROS-induced damage, HIF-1α interacts with NRF2, enhancing its activation and promoting the expression of NRF2 target genes such as glutathione S-transferase (GST) and NAD(P)H quinone dehydrogenase 1 (NQO1), which aid in ROS detoxification. NRF2, in turn, activates downstream AREs, including HO-1 [46], SOD [47], glutathione peroxidases (GPx) [48], NADPH oxidases (NOX) [49,50,51] (Figure 3). In turn, NRF2 modulates the expression of HIF-1α target genes [52], creating a feedback loop that strengthens the antioxidant response. The release of mitochondrial ROS of neutrophils is elevated, and the elevated ROS further enhance the stabilization of HIF1α in hypoxia [53]. This cross-talk between HIF and NRF2 helps cells adapt to oxidative stress and prevent damage during hypoxia.

The HIF pathway helps cells respond to hypoxia and regulates the antioxidant response to protect against oxidative damage. Under normal oxygen levels (normoxia), HIF is quickly degraded. However, under hypoxic conditions, the enzymes that degrade HIF are inhibited, allowing HIF-α to stabilize, enter the nucleus, and bind to DNA with HIF-β. This activates genes involved in hypoxia adaptation and oxidative stress regulation. Chronic hypoxia increases ROS production by upregulating NOX and activating of NRF2, and HIF activation promotes antioxidant defenses by upregulating genes such as HO-1, SOD, GPx, and NOX to counteract ROS damage.

#### 2.3.3. HIF Modulates ROS Production and Promotes Cancer Progression

HIF also modulates ROS production [18,54]. Under normoxic conditions, mitochondria are the primary producers of ROS. However, during acute hypoxia, HIF induces a metabolic shift towards glycolysis (the Warburg effect), which reduces mitochondrial ROS production (Figure 3). Hypoxia induces the lactylation of mitochondrial proteins PDHA1 and CPT2 and restricts oxidative phosphorylation, thereby reducing the mitochondrial ROS [55]. This shift not only limits ROS generation but also helps maintain redox balance by upregulating key enzymes involved in NADPH production, which supports the antioxidant system [56,57].

In cancer, the HIF pathway is often activated due to the rapid growth of the tumor, leading to hypoxic microenvironments. Tumor cells rely on HIF to adapt to hypoxia and oxidative stress, enhancing their antioxidant defenses. This enables the survival of cancer cells in low-oxygen, high-ROS environments, contributing to tumor progression, resistance to therapy, and metastasis. Moreover, the HIF-mediated upregulation of antioxidant genes contributes to tumor cell resistance to chemotherapy and radiation, both of which rely on ROS generation to trigger cell death. HIF-1α regulates the expression of enzymes involved in maintaining the cellular redox state, including lactate dehydrogenase (LDH) and pyruvate kinase M2 (PKM2), which support glycolysis and help maintain cellular ATP levels under hypoxic conditions. HIF-1α upregulated PKM2 by activating ALYREF and leads to poor survival in bladder cancer patients [58]. Under hypoxia, the long noncoding RNA AC020978 promotes the nuclear translocation of PKM2, regulates PKM2-enhanced HIF-1α transcription activity and indicates an aggressive phenotype of non-small cell lung cancer (NSCLC) [59]. HIF-1 also plays a role in protecting cells from oxidative DNA damage [60]. HIF-1α induces YAP nuclear translocation, and YAP protects cells from DNA damage [60]. Moreover, the HIF-1α/miR-210/Rad52 pathway participates in nano-Ni-induced DNA damage and contributes to genotoxicity and carcinogenicity [61].

### 2.4. Tumor Suppressor p53 Pathway

**Figure 4:**
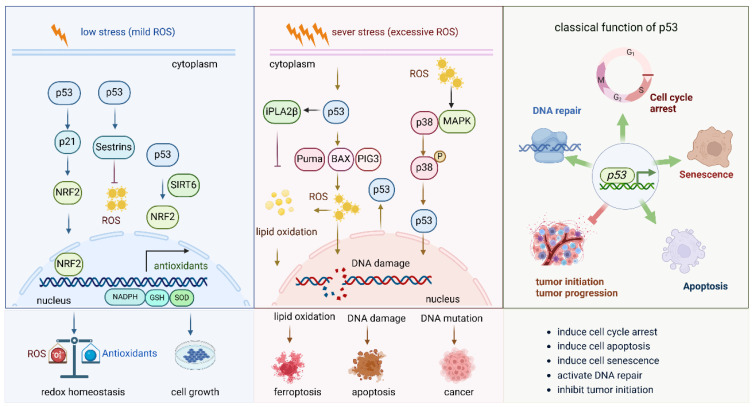
Role of P53 pathway in oxidative stress.

The tumor suppressor protein p53, known as the “guardian of the genome”, plays a pivotal role in regulating cellular responses to stress, particularly oxidative stress, by orchestrating key processes such as cell cycle arrest, apoptosis, and DNA repair. In response to oxidative damage, p53 becomes activated and plays a pivotal role in maintaining cellular integrity (Figure 4).

This figure illustrates the role of p53 under mild oxidative stress and severe oxidative stress. (1) Under mild oxidative stress, p53 activates the Nrf2 pathway by different pathways, such as the p53-p21-NRF2 pathway and the p53-sestrins-Nrf2 pathway. It also cooperates with SIRT6 to activate the NRF2 pathway. Activated NRF2 induces the expression of a series of antioxidant enzymes to eliminate the oxidative stress and keep the redox balance of the cell. (2) Under sever oxidative stress, the activated p53 induces cell death via different molecules. By inducing the expression of PUMA, BAX, and PIG3, it promotes ROS production and induces DNA damage; the increased ROS levels further lead to lipid oxidation and finally to cell ferroptosis. DNA damage and DNA mutation under high oxidative stress lead to cell apoptosis and cancer initiation, respectively. (3) The general and classical functions of p53 in biological and pathological circumstances.

#### 2.4.1. P53 Boots Antioxidant Defense Capacity Under Mild Oxidative Stress

Under low stress (mild ROS), p53 enhances the expression of the Nrf2 pathway, which further boosts the antioxidant defense capacity of the cell. P53 activates the Nrf2 pathway via the p53-p21-Nrf2 pathway [62], in which p21 interacts with Nrf2. In addition, sestrins are among the p53 targets, are oxidative stress-sensing proteins and have stress-suppressive functions. Activation of p53 also activates Nrf2 via upregulating sestrins [63]. p53 enhances the transcriptional activity of SIRT6, which then functions as a co-transcriptional regulator and a key docking molecule, bridging p53 and Nrf2 to mitigate oxidative stress and promote hepatocyte proliferation [64]. By activating the Nrf2 signaling pathway, p53 promotes the transcription of genes encoding antioxidants and detoxifying enzymes, such as GST, NQO1, and catalase, which help to neutralize ROS and prevent cellular damage. In this way, p53 not only regulates cell cycle checkpoints and DNA repair but also helps initiate a protective response against oxidative stress, ensuring cellular homeostasis and promoting survival under conditions of oxidative damage. This interaction between p53 and antioxidant defense pathways plays a critical role in preventing the accumulation of cellular damage that could otherwise lead to tumorigenesis or cellular senescence.

#### 2.4.2. P53 Induces Cell Death Under Severe Oxidative Stress

Under severe oxidative stress (excessive ROS), p53 activation increases ROS production, especially from mitochondria [65]. Simultaneously, p53 induces the expression of genes such as p21, which causes cell cycle arrest, and SOD, which reduces ROS levels, helping to repair oxidative DNA damage. This maintains a balance between cellular damage and repair. If damage is excessive, p53 activates pro-apoptotic proteins, including PUMA, which induces BAX, resulting in mitochondrial outer membrane permeabilization and apoptosis. ROS further amplify this pathway by increasing mitochondrial permeability. Additionally, p53-induced gene 3 (PIG3), upregulated by p53, enhances ROS production, contributing to DNA damage and promoting apoptosis [66]. p53 also activates the p38-MAPK pathway, which regulates inflammation, DNA repair, and survival. p38 cooperates with p53 to induce cell cycle arrest or apoptosis under stress. If p53 fails to eliminate damaged cells, mutations in p53 or other stress response genes may lead to the survival of genetically unstable cells, contributing to cancer. Furthermore, oxidative stress and p53 activation induce lipid peroxidation and ferroptosis, with calcium-independent phospholipase A2β (iPLA2β) suppressing p53-driven ferroptosis under stress [67]. Lipid peroxides accumulate, promoting ferroptosis. Thus, p53 maintains a balance between promoting survival through repair and inducing cell death by apoptosis, DNA mutation, or ferroptosis, depending on damage severity. Failure in this regulation, often due to p53 mutations, leads to cancer and resistance to cell death, further complicating the cellular response to stress.

### 2.5. PI3K-Akt Pathway

**Figure 5:**
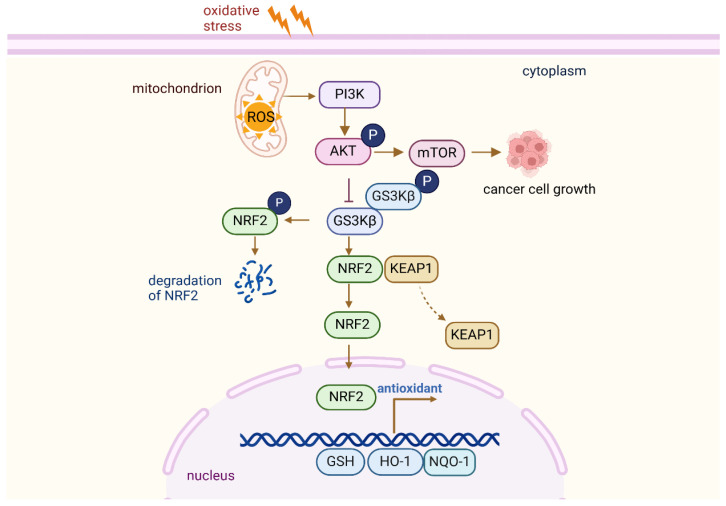
PI3K-Akt pathway regulates antioxidant responses.

The PI3K-AKT pathway plays a crucial role in cellular signaling, regulating cell growth, survival, metabolism, and antioxidant responses [68]. Its activation under oxidative stress is essential to the cellular adaptation mechanisms that protect against damage caused by ROS [10]. Upon activation, AKT phosphorylates a variety of downstream targets involved in cell survival, apoptosis inhibition, and metabolic regulation. A crucial downstream function of AKT under oxidative stress is the regulation of antioxidant proteins. AKT activation indirectly modulates the activity of several transcription factors involved in the antioxidant responses. AKT, through the mTORC1 complex, phosphorylates and activates components of the mTOR pathway, which in turn regulates the expression of antioxidant genes through NRF2. The phosphorylation of glycogen synthase kinase 3β (GSK-3β), a key inhibitor of NRF2, by AKT inhibits its activity, preventing the degradation of NRF2 and allowing its stabilization [69]. As a result, NRF2 translocates to the nucleus, binds to ARE, and induces the transcription of a wide range of antioxidant genes, including superoxide dismutase (SOD), catalase, and glutathione peroxidase (GPx). These enzymes play a direct role in mitigating oxidative damage by neutralizing ROS, thereby safeguarding the cells from oxidative stress (Figure 5).

In addition to modulating NRF2, the PI3K-AKT pathway also influences other key players in redox signaling. AKT activation increases the expression of thioredoxin and thioredoxin reductase, which contribute to maintaining the redox balance within the cell [70]. Moreover, the PI3K-AKT pathway is crucial in the regulation of mitochondrial function, which is essential for the cellular response to oxidative stress. By promoting mitochondrial biogenesis and regulating mitochondrial dynamics, AKT ensures that the cellular energy supply remains intact even under oxidative stress. This, in turn, supports the proper functioning of antioxidant systems and minimizes oxidative damage to cellular components, including DNA, proteins, and lipids. The activation of the PI3K-Akt-mTOR pathway inhibits ferroptosis by SREBP-mediated lipogenesis [71].

The PI3K-AKT pathway does not operate in isolation but is interconnected with other signaling pathways that contribute to the antioxidant response. For instance, AKT interacts with the AMP-activated protein kinase (AMPK) pathway, which also functions in cellular defense against oxidative stress [72]. The cross-talk between these pathways amplifies the cellular capacity to counteract oxidative damage and maintains cellular homeostasis under stress conditions. Furthermore, AKT-induced regulation of autophagy, also supports antioxidant defenses by preventing the accumulation of ROS-generating dysfunctional components [73]. In cancer, the aberrant activation of the PI3K-AKT pathway not only promotes cell survival and growth but also enhances the antioxidant defense mechanisms, contributing to tumor progression and resistance to therapies that induce oxidative stress, such as chemotherapy and radiation. In conclusion, the PI3K-AKT signaling pathway plays a central role in regulating cellular antioxidant responses through the activation of NRF2 and other redox-related proteins. By controlling the expression and activity of antioxidant enzymes, the PI3K-AKT pathway helps maintain cellular redox homeostasis and protects against oxidative damage.

The interaction between AKT, GSK3β, and NRF2 in cellular stress response. Upon activation, AKT phosphorylates and inhibits GSK3β, preventing its degradation of NRF2. This leads to the stabilization and accumulation of NRF2, which then translocates to the nucleus to initiate the transcription of antioxidant and detoxifying genes. This pathway plays a key role in regulating cellular responses to oxidative stress and promoting cell survival.

### 2.6. NF-kB and Antioxidants

The canonical NF-κB pathway is mainly activated by the stimulation of proinflammatory receptors, including those in the TNF receptor superfamily, cytokine receptors for interleukins, and Toll-like receptor family (TLRs). NF-κB contributes to the sustained inflammatory environment, cell survival, proliferation, and metastatic potential of cancer cells. Under oxidative stress, NF-κB serves as a dual-function pathway: it helps protect cells from oxidative stress, but it also amplifies oxidative stress via inflammation and increased ROS production. This duality is context-dependent and varies depending on the duration and intensity of the oxidative stress.

**Figure 6:**
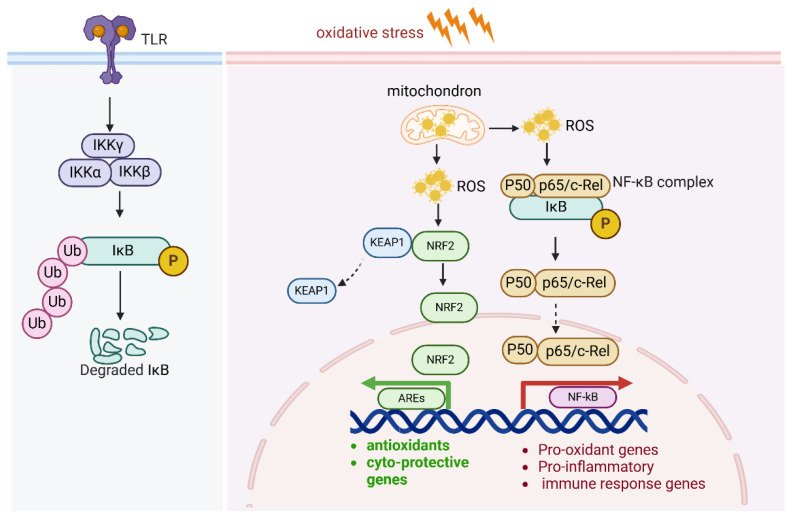
NF-κB pathway and antioxidants.

ROS activate the IKK complex, which is a central regulator in NF-κB activation. The IKK complex is composed of three subunits: IKKα, IKKβ, and IKKγ (NEMO). In the resting state, NF-κB dimers (commonly p65/p50) are kept inactive in the cytoplasm by binding to inhibitory proteins, primarily IκBα. ROS cause modifications in IKKβ, such as phosphorylation and oxidation of cysteine residues, which activates the IKK complex. Upon activation, IKKβ phosphorylates IκBα at specific serine residues (Ser32 and Ser36), resulting in its ubiquitination and proteasomal degradation. This degradation frees the NF-κB dimers from their inhibitory complex, allowing them to translocate into the nucleus. In the nucleus, the active NF-κB dimers (such as p65/p50) bind to κB sites in the promoters of target genes, initiating the transcription of pro-inflammatory cytokines, survival factors, and genes involved in cell proliferation, immune response, and apoptosis regulation (Figure 6). Furthermore, ROS also activate upstream kinases that enhance IKK activation, such as apoptosis signal-regulating kinase 1(ASK1). ROS activate MAPKs and PI3K/Akt, which further amplify NF-κB activation.

Under oxidative stress, NF-κB serves as a dual-function pathway: it helps protect cells from oxidative stress, but it also exacerbates oxidative stress via inflammation and increased ROS production. ROS activate the IKK complex and NF-kB pathway. In addition, NF-κB activity also regulates Nrf2-mediated ARE expression. The subunit of NF-kB p65 negatively regulates AREs by enhancing KEAP1 expression.

### 2.7. Epigenetic Regulation of Antioxidant Genes

In addition to classical signaling pathways, epigenetic mechanisms play a crucial role in modulating antioxidant gene expression. DNA methylation of promoter regions can lead to the silencing of key antioxidant enzymes such as *SOD2*, *GPX3*, and *PRDX1*, especially in tumor cells under persistent oxidative stress. For instance, hypermethylation of the *GPX3* promoter has been observed in prostate and gastric cancers, correlating with reduced expression and poor prognosis [74]. Histone modifications, such as H3K27me3 and H3K9ac, also influence chromatin accessibility of redox-regulating genes. The histone methyltransferase EZH2, often upregulated in cancers, has been shown to repress NRF2 target genes by promoting H3K27 trimethylation [75]. These epigenetic alterations are reversible and represent potential therapeutic targets to restore antioxidant defenses in the tumor microenvironment.

## 3. The Dual Function of Antioxidants in Cancer Therapy

Antioxidants are widely recognized for their protective roles in cellular biology, primarily through their ability to neutralize ROS and reduce oxidative stress. However, in cancer therapy, antioxidants exhibit a paradoxical behavior. Depending on the biological environment, antioxidants have pro-oxidant effects that either facilitate tumorigenesis or contribute to therapy resistance.

### 3.1. Pro-Oxidant Effect of Antioxidants

While antioxidants are typically associated with protecting cells from oxidative damage, under certain conditions, they exacerbate oxidative stress, thereby enhancing tumorigenesis or promoting resistance to cancer treatments. Some antioxidants, such as vitamin C or N-acetylcysteine (NAC), have been shown to act as pro-oxidants when administered at high concentrations, thereby enhancing the production of ROS within tumor cells [76]. The pro-oxidative activity of vitamin C is primarily dependent on the availability of iron. Ascorbate reduces iron to its ferrous (Fe^2+^) state, which readily reacts with oxygen. This reaction, part of the Fenton reaction, produces ROS and hydrogen peroxide (H_2_O_2_). The H_2_O_2_, in turn, reacts with Fe^2+^ to generate highly reactive hydroxyl radicals [76]. This unintended increase in ROS paradoxically activates oncogenic signaling pathways such as NF-kB and MAPK, which are involved in cell proliferation, survival, and metastasis.

Moreover, antioxidants facilitate resistance to chemotherapy and targeted therapies through modulating cellular redox states. They mitigate the cytotoxic effects of chemotherapeutic agents by scavenging the ROS generated by the drugs, thereby preventing ROS-induced DNA damage. This has been observed in drugs such as doxorubicin and cisplatin, where elevated antioxidants protect tumor cells from oxidative damage, allowing them to survive and proliferate despite treatment [77]. Additionally, antioxidants reduce ROS levels, which in turn diminish the activation of key pro-apoptotic pathways, thus enhancing cell survival and contributing to the overall resistance of tumors to therapeutic intervention [78].

### 3.2. Antioxidants as Chemo- and Radio-Sensitizers

Despite their potential to act as pro-oxidants, antioxidants also function in sensitizing tumors to chemotherapy and radiotherapy [79]. Chemotherapy and radiotherapy exert their therapeutic effects primarily through the induction of ROS, which inflict oxidative damage to DNA, proteins, and lipids, ultimately resulting in cell death. The efficacy of these treatments is, therefore, closely tied to the cellular redox status. Antioxidants enhance the therapeutic efficacy of chemotherapy and radiotherapy by modulating the ROS balance and promoting cellular responses that favor the therapeutic outcomes [80]. For instance, antioxidants such as vitamin E and curcumin potentiate the effects of certain chemotherapeutic agents via increasing ROS levels and enhancing oxidative stress within tumor cells [81]. The combination of NAC with chemotherapy agents such as Paclitaxel has been found to increase ROS production, leading to enhanced tumor cell death [82]. Similarly, antioxidants also increase the radio-sensitivity of tumors by inhibiting the repair of radiation-induced DNA damage, thus promoting tumor cell death through enhanced oxidative damage [83]. Furthermore, antioxidant metabolism modulates the immune system to enhance the efficacy of cancer therapies. By modulating ROS-induced immune responses, antioxidants may facilitate anti-tumor immunity, which complement the direct cytotoxic effects of chemotherapy and radiotherapy. This has been demonstrated in preclinical studies, where antioxidant treatment was associated with improved immune cell infiltration and enhanced tumor cell recognition by immune cells. For example, antioxidants such as resveratrol and melatonin have been shown to modulate the TME and enhance the activity of immune checkpoint inhibitors, potentially improving the therapeutic outcomes of immunotherapy in combination with conventional treatments [84,85,86]. Antioxidant metabolism itself can be targeted to improve CAR T cell therapy. The FDA-approved IDH2 inhibitor Enasidenib boosts memory CAR T cell formation and maintains anti-leukemic efficacy by reducing oxidative stress and limiting histone acetylation [87]. This approach improves CAR T cell function in pre-clinical models, overcoming exhaustion and enhancing therapy outcomes.

### 3.3. Antioxidants in the Tumor Microenvironment

The TME is a dynamic and intricate network consisting of tumor cells, stromal cells, extracellular matrix, and soluble factors that contribute to cancer progression, metastasis, and resistance to therapy. Antioxidants play a vital role in modulating various components of the TME, including tumor–stroma interactions, angiogenesis, immune evasion, and the extracellular matrix, thereby influencing tumor progression and response to therapy [88,89].

#### 3.3.1. Antioxidants Impact Tumor–Stroma Interactions

Antioxidants impact tumor–stroma interactions by altering the redox status of both cancer cells and surrounding stromal cells, including endothelial cells, fibroblasts, and immune cells. The oxidative stress in TME drives fibroblast activation and kidney fibrosis [90]. Antioxidants influence the production of cytokines and growth factors that promote angiogenesis [23]. By modulating the redox signaling pathways in endothelial cells, antioxidants either enhance or inhibit the formation of new blood vessels, thereby affecting tumor growth and metastasis. Antioxidants such as SOD mimetics and catalase reduce oxidative stress in the TME, potentially slowing down angiogenesis and limiting the supply of nutrients and oxygen to tumors [91]. Conversely, antioxidants may also promote angiogenesis in certain contexts, depending on the balance of ROS within the TME. Cancer-associated fibroblast-secreted mirR-522 serves as an antioxidant to suppress ROS production and promotes gastric cancer chemo-resistance [92].

#### 3.3.2. Antioxidants Influence the Immune Response

Antioxidants influence the immune response within the TME, which plays a critical role in immune evasion and tumor progression. ROS are key regulators of immune cell function, including the activation of T cells and macrophages [93,94]. The use of responsive lipid nanoparticles for targeted delivery of vitamin C to tumor sites effectively induces oxidative damage and drives M1 macrophage polarization. This, in turn, enhances the infiltration of activated cytotoxic T lymphocytes into the tumor microenvironment, supporting the effectiveness of immune checkpoint blockade therapy [95]. By modulating ROS levels, antioxidants either enhance immune responses or promote immune suppression. In certain cancers, antioxidants reduce oxidative stress within immune cells, resulting in impaired immune surveillance and the suppression of anti-tumor immunity. On the other hand, in cancers with an already inflamed TME, antioxidants may help restore immune function and promote the anti-tumor activity of immune cells.

#### 3.3.3. Antioxidants Modulate the Extracellular Matrix

Antioxidants play a vital role in modulating the extracellular matrix (ECM) within the TME, influencing cancer progression and therapeutic outcomes [96]. Novel antioxidants target ECM remodeling to enhance cancer treatment efficacy. Flavonoids, such as Apigenin, reduce ECM deposition and preserve collagen integrity [97]. Apigenin inhibits ECM production in transforming growth factor-beta 1 (TGF-β1)-stimulated cardiac fibroblasts by modulating specific signaling pathways, which may have implications for tumor-associated fibrosis [98]. Additionally, innovative synthetic antioxidants have been developed to selectively target tumor ECM components [99]. These compounds exhibit potent anticancer activity by disrupting the structural integrity of the ECM, thereby inhibiting tumor growth and metastasis. Furthermore, the interaction between antioxidants and ECM stiffness has been investigated in cancer therapy [100]. Oxidative stress can induce covalent crosslinking of ECM proteins such as collagen and elastin via ROS-mediated reactions, leading to increased ECM stiffness. This stiffened ECM alters cell-ECM interactions and promotes a more invasive tumor phenotype. Antioxidants counteract this effect by reducing ROS levels and inhibiting the activity of matrix crosslinking enzymes such as lysyl oxidase (LOX), which catalyzes collagen crosslinking. As a result, antioxidants may help preserve a more compliant ECM structure, reducing stiffness and potentially suppressing cancer cell invasion, immune evasion, and drug resistance. Modulating ECM stiffness through antioxidant treatment can influence tumor progression, cancer immunity, and drug resistance, offering potential therapeutic strategies.

## 4. Antioxidant Strategies Exploration

**Figure 7:**
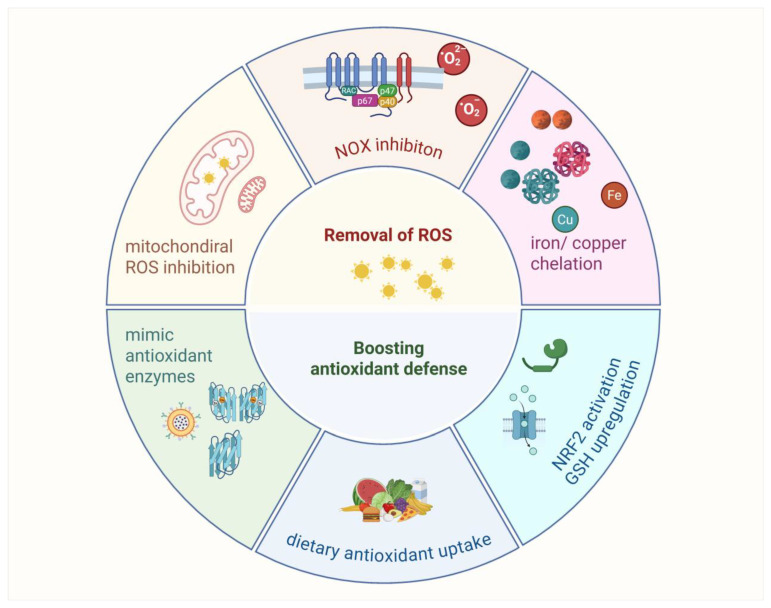
Antioxidants strategy exploration.

Various antioxidant strategies are being explored in clinical trials, including removing ROS, eliminating H_2_O_2_ before it becomes •OH or HOX, boosting GSH levels with precursors, increasing antioxidant enzyme production through NRF2 activation, inhibiting NOXs, enhancing mitochondrial antioxidant defense, supplementing with dietary antioxidants, and inhibiting abnormal redox signaling (Figure 7).

Antioxidant strategies in cancer therapy focus on reducing oxidative stress to enhance treatment efficacy. Approaches include removing ROS with mitochondrial-targeted antioxidants such as OP2113 and inhibiting NADPH oxidases to limit excessive ROS production. Chelation therapy targets iron and copper to prevent hydroxyl radical formation, though its broader use is limited by the essential roles of these metals. Boosting antioxidant defenses involves enhancing GSH levels with precursors such as NAC or activating NRF2 signaling to upregulate antioxidant enzymes. Additionally, enzyme mimics such as Mn porphyrins and GPX analogs show potential in reducing oxidative damage. These strategies offer promising avenues for improving cancer therapies, though their clinical applications require further validation.

### 4.1. Removal of ROS

Removal of ROS involves neutralizing ROS (O_2_•− and H_2_O_2_) before they interact with other molecules to form harmful compounds such as ONOO− or HOX [101]. There are mainly two ways sources of ROS: main ROS from mitochondria in the process of oxidative phosphorylation and others originating from the NADPH oxidase pathway.

#### 4.1.1. Mitochondrial ROS Production Inhibition

Mitochondrial antioxidant defenses are important, as mitochondria produce ROS in ETC [8,102]. While inhibiting ROS production directly in mitochondria is challenging due to potential disruptions in ATP production, compounds such as OP2113 have been proposed to specifically inhibit complex I ROS production without affecting ATP [103]. Additionally, SOD mimics have been developed to enhance H_2_O_2_ production in mitochondria, but these have toxic effects, such as the compound Ebselen [104]. Antioxidants targeted to mitochondria using lipophilic cations are under development [105].

#### 4.1.2. NADPH Oxidase Inhibition

NADPH oxidases (NOXs) play a crucial role in redox signaling and immune function but cause tissue damage when overactivated. Two main strategies are being explored: direct inhibition of NOX enzyme activity (e.g., with diphenyleneiodonium and apocynin) and prevention of NOX complex assembly, particularly for NOX2 [106]. While some NOX inhibitors and small peptides such as Ebselen, CYR5099, apocynin and GKT137831 target NOX assembly, no such drugs have yet advanced to clinical use.

#### 4.1.3. Chelation of Iron and Copper Therapy

Iron and copper are important metals in the body, but when released from proteins, they promote the production of harmful hydroxyl radicals (•OH), which cause oxidative damage [107]. Chelating agents reduce iron level by binding to iron and copper, preventing them from contributing to the production of these radicals [108]. However, challenges remain, since iron and copper are essential for many biological functions, including energy production and DNA synthesis. Therefore, chelation therapy is generally only used in cases of metal overload, such as sickle cell disease or thalassemia, where frequent blood transfusions can lead to excessive iron accumulation [109]. In these cases, chelation helps prevent iron toxicity and related organ damage. However, using chelators to generally remove iron or copper for controlling ROS production is complicated. The key is to develop chelators that specifically target excess iron and copper without disrupting the body’s normal processes [110]. With further advancements, chelation therapy could become a more effective tool in managing oxidative damage while preserving essential metal functions.

### 4.2. Boosting Antioxidant Defenses

Boosting antioxidant defenses can be achieved by increasing levels of glutathione (GSH) through precursors, activating antioxidant enzymes via NRF2, and supplementing with dietary antioxidants such as vitamins C and E.

#### 4.2.1. Development of Antioxidant Enzyme Mimics

SOD is critical for neutralizing O_2_•− and protecting •NO, making it a key defense against oxidative stress. Mimics such as nitroxides, Mn cyclic polyamines, metalloporphyrins, Mn–salen complexes, and fullerenes have been developed, with early studies focusing on metalloporphyrins. While these mimics are less effective than natural enzymes in the cytosol, they show promise in extracellular spaces with low antioxidant enzyme concentrations and might work in mitochondria, although some act as pro-oxidants [111]. Most SOD mimics, developed to target O_2_•−, also reduce other reactive species, such as ONOO−, H_2_O_2_, and CO^3^•− [112]. Some, such as Mn porphyrins, act as pro-oxidants, interfering with redox-sensitive signaling. While SOD itself, developed as the drug orgotein, has shown promise in reducing radiation therapy side effects, it has not been approved for human use. Mn porphyrins, such as MnTE-2-pYp^5+^, have shown therapeutic effects in animal models for diseases such as stroke and cancer [113]. Another SOD mimetic, GC4419, selectively targets superoxide anions and has shown potential in clinical trials [114]. Ebselen, a GPX mimic, has demonstrated effectiveness in reducing oxidative damage and inflammation in animal studies and is in clinical trials for conditions such as Meniere’s disease and stroke [115]. A newer GPX mimic, ALT-2074, has shown potential in reducing inflammation and oxidative damage in animal models and is undergoing trials for diabetes and coronary artery disease [116]. While these mimics are less effective than natural enzymes in the cytosol, they are promising for use in areas with low natural antioxidant enzyme concentrations. Some compounds, such as Mn porphyrins, also have pro-oxidant effects, and research continues to assess their safety and therapeutic potential.

#### 4.2.2. Dietary Antioxidants Uptake

Dietary antioxidants, especially vitamin C (ascorbic acid) and vitamin E (α-tocopherol), are commonly used to mitigate oxidative stress [117,118]. Both vitamins play essential roles in neutralizing free radicals and protecting cells from oxidative damage. However, while these antioxidants are generally beneficial, evidence of their therapeutic effectiveness is inconsistent, with some studies showing benefits for conditions such as cardiovascular disease and cancer [119], while others suggest they may promote cancer development. This dual nature of antioxidants, including their role in redox signaling, requires further research, as they can sometimes enhance signaling pathways that may be detrimental, such as in the case of cancer progression.

#### 4.2.3. NRF2 Pathway Activation and GSH Upregulation

NRF2 activators, found in substances such as polyphenols and dietary supplements, function by modulating proteins, including KEAP1, or inhibiting BACH1, thereby triggering the NRF2 pathway [120]. This approach is under clinical investigation for a range of conditions, such as chronic obstructive pulmonary disease (COPD), osteoarthritis, and diabetic nephropathy [16]. Although certain NRF2 activators have shown promise, several challenges persist, particularly regarding their low bioavailability and the potential for nonspecific effects, which may disrupt other cellular signaling pathways. Furthermore, prolonged activation of NRF2 may pose significant risks, including the promotion of cancer cell growth or the development of resistance to chemotherapy [121].

In parallel, strategies aimed at increasing intracellular GSH levels, such as NAC and GSH esters, are being investigated. NAC and GSH esters serve to replenish cellular GSH, with GSH esters demonstrating enhanced efficiency in cellular delivery. However, the clinical efficacy of these approaches remains uncertain, as the results from animal models and clinical trials have been inconsistent.

## 5. Antioxidants in Clinical Cancer Trials

### 5.1. Overview of Preclinical Studies

Antioxidants sensitize tumors to chemotherapy and radiation or reduce tumor growth by modulating the oxidative microenvironment (Table 1). NAC reduces oxidative stress in animal models of leukemia and breast cancer, increasing chemotherapy efficacy. Additionally, sulforaphane, an antioxidant derived from cruciferous vegetables, has exhibited anti-cancer effects in preclinical studies by inhibiting tumor growth and metastasis in breast cancer xenografts [122]. In breast cancer xenografts, sulforaphane, a natural antioxidant derived from cruciferous vegetables, has been shown to inhibit tumor growth and metastasis by downregulating matrix metalloproteinases and epithelial–mesenchymal transition (EMT) markers [122]. Likewise, resveratrol, a polyphenol antioxidant, demonstrated synergistic effects with doxorubicin in breast and prostate cancer models by enhancing ROS-mediated apoptosis and suppressing angiogenesis [123]. Antioxidants such as curcumin and quercetin have also been found to sensitize tumors to radiation therapy in models of glioblastoma and head and neck cancers by inhibiting the NF-κB signaling pathway and reducing DNA repair activity [124]. Furthermore, ascorbic acid (vitamin C) has shown promise in colorectal and pancreatic cancer models, where high pharmacologic doses generate pro-oxidant effects that are selectively toxic to cancer cells. On the other hand, antioxidants may interfere with the cytotoxic effects of chemotherapy and radiotherapy. For instance, vitamin E has been found to reduce ROS generation in colorectal cancer models, ultimately impairing the effectiveness of oxaliplatin-based chemotherapy. Similarly, glutathione supplementation has been associated with enhanced tumor cell survival and accelerated tumor growth in melanoma models.

### 5.2. Clinical Trials and Observations

Clinical trials examining antioxidants in cancer therapy have yielded mixed results. The focus has primarily been on antioxidants’ ability to alleviate treatment-induced side effects (e.g., chemotherapy-induced neuropathy, fatigue) and their potential to enhance the effectiveness of chemotherapy, radiotherapy, and immunotherapy. Clinical trials have explored the use of antioxidants such as NAC, vitamin C, and selenium, in combination with chemotherapy and radiotherapy [125]. Selenium, an essential trace element with antioxidant properties, contributes to redox balance by being incorporated into selenoproteins such as glutathione peroxidases [126]. Clinically, selenium supplementation has been studied in cancer patients, including those with head and neck or gastrointestinal cancers, where it showed potential in reducing treatment-related toxicities and improving quality of life. In addition, Melatonin acts as a potent free radical scavenger by directly neutralizing reactive oxygen and nitrogen species and upregulating antioxidant enzymes such as superoxide dismutase and glutathione peroxidase [127]. Clinically, melatonin has been evaluated in trials for patients with solid tumors (e.g., breast and prostate cancer), where it was shown to reduce chemotherapy-induced toxicity and improve overall treatment tolerance [128].

NAC has been applied in clinical trials in the treatment of excoriation disorder [129], psychiatry and neurology [130], aging [131], systemic lupus erythematosus disease [132], acute organophosphorus pesticide poisoning [133], and idiopathic pulmonary fibrosis [134]. Antioxidants such as vitamin C and melatonin have also been evaluated, as they can regulate the immune response in cancer patients. High-dose vitamin C impacts the infiltration of immune cells into the TME and slows tumor growth through a T cell-dependent mechanism. Vitamin C not only enhances the cytotoxic activity of adoptively transferred CD8 T cells but also works synergistically with immune checkpoint therapy (ICT) across multiple cancer types. The pairing of vitamin C with ICT has shown potential in achieving therapeutic benefits in models of tumors characterized by mismatch repair deficiency and a high mutational burden [135]. Several antioxidants have been specifically investigated in pre-clinical settings for their effects on cancer therapy outcomes (Table 1).

Despite promising preclinical evidence, the clinical translation of antioxidants in cancer therapy faces several challenges. These include the difficulty in identifying appropriate patient subgroups, determining optimal dosing regimens, and avoiding interference with standard treatments. Furthermore, discrepancies in trial outcomes may stem from variations in tumor type, treatment timing, or antioxidant form (e.g., oral vs. intravenous vitamin C). Ongoing and future trials are increasingly focused on biomarker-guided approaches, such as redox status or immune signatures, to identify patients who may benefit most from antioxidant co-therapy. This precision-guided use of antioxidants may ultimately resolve current controversies and improve therapeutic outcomes in selected cancers.

**Table 1:** Typical antioxidants in cancer treatment.

Antioxidant	Key Findings	Study Type
Vitamin C	(1) As an adjunct to chemotherapy in pancreatic cancer [136]. (2) Enhanced glioblastoma progression [137]. (3) A complementary strategy to traditional therapy in breast cancer [138]. (4) Inhibited ovarian cancer metastasis [139].	Pre-clinical study
N-Acetylcysteine	(1) Protection from lung emphysema but induction of lung cancer in mice [140]. (2) Overcoming NF1 loss-driven resistance to PI3Kα inhibition in breast cancer [141]; exhibiting anti-proliferative effect in breast cancer [142]. (3) Protects against cisplatin-induced cognitive impairments in ovarian cancer [143]. (4) Reduces the risk of hepatocellular carcinoma [144,145].	Pre-clinical study
Sulforaphane	(1) Induced cell death in pancreatic cancer [146]; inhibited cancer progression without side effects [147]. (2) Inhibited lung cancer stem cell renewal [148]. (3) Inhibited bladder cancer metastasis by blocking actin nucleation-driven pseudopodia formation [149]. (4) Improved glioblastoma treatment, caused cell apoptosis [150].	Pre-clinical study
Vitamin E	(1) Higher serum alpha-tocopherol levels and the correction of low vitamin E status were associated with a reduced risk of lung cancer over a 28-year period [151]. (2) The clinical trial on selenium and vitamin E for bladder cancer recurrence (NO. ISRCTN13889738) revealed that vitamin E supplementation could be harmful to patients [152].	Pre-clinical study and clinical trial
Glutathione	(1) GPX4 degradation enhances ferroptosis-induced anti-tumor immunity in pancreatic cancer [153]. (2) Suppressing de novo glutathione synthesis overcomes acquired resistance to EGFR-targeted therapy in lung cancer [154]. (3) GPX4 prevents ferroptosis and contributes to tamoxifen resistance in breast cancer [155]. (4) Induction of ferroptosis and inhibition of glutathione is a therapeutic approach in prostate cancer [156].	Pre-clinical study
Melatonin	Melatonin effectively reduces oxidative stress through direct detoxification of reactive species and by modulating enzyme activity to enhance antioxidants and suppress pro-oxidants [86], it inhibits cancer progression [157,158].	Pre-clinical study
Selenium	Selenium detoxification is essential for cancer cell survival [126]; while organic selenium induces pancreatic cancer cell ferroptosis [159], selenium nanoparticles (SeNPs) show promise in lung cancer treatment by inhibiting cancer proliferation, enhancing immunity, and improving therapeutic efficacy while offering diagnostic benefits [160]. Selenium and vitamin E have been applied in clinical trials for the treatment of non-muscle-invasive bladder cancer [152].	Pre-clinical study and clinical trial
Curcumin	It has antioxidant, anti-inflammatory and anti-cancer properties [161,162]. Curcumin modulates drug sensitivity in NSCLC by influencing the cell cycle, MAPK, NF-kappa B, and Th17 cell-differentiation signaling pathways [163]. Three studies focused on the use of minimal curcumin and vitamin D in patients with leukemia (NCT02100423), cervical/uterine cancer (NCT03192059), and pancreatic cancer (NCT02336087).	Pre-clinical and clinical trial
Alpha-lipoic Acid	(1) It regulates prostate cancer cell growth and bone cell differentiation [164], while also inhibiting cell proliferation, suppressing autophagy, and counteracting prostate cancer cell migration [165]. (2) It inhibits lung cancer growth by mTOR-mediated autophagy inhibition [166]. (3) It targets KLF7 to inhibit cervical cancer progression [167].	Pre-clinical study on cancer and clinical trial in non-cancer diseases
Resveratrol	It is a phytoalexin antioxidant that has been proven to have anticancer effects in various cancers [168]. Resveratrol inhibits malignant phenotypic alterations that drive cell migration and drug resistance, thereby enhancing colorectal cancer treatment by interacting with p53 [169].	Pre-clinical study and clinical trial
Green tea extract (EGCG)	It prevents prostate cancer progression and induces lung cancer cell apoptosis [170,171]. It also shows anticancer potential on breast cancer spheroids [172].	Pre-clinical and clinical trial
Coenzyme Q10	Coenzyme Q10, as an antioxidant, plays a role in targeting the pathways involved in BC tumor progression [173].	Clinical trial
Pterostilbene (PTE)	It is an active compound extracted from blueberries and grapes. (1) PTE treatment independently inhibited cell proliferation and viability while inducing apoptosis by targeting COX2 in lung cancer [174]. (2) PTE inhibited breast cancer metastasis by the NF-kB pathway [175]. (3) PTE inhibited irradiation-resistant glioma stem cells by modulating the GPR78/miR-205 axis. (4) PTE enhanced the sensitivity of cisplatin-resistant human bladder cancer cells harboring oncogenic HRAS [176].	Pre-clinical study and clinical trial
Lycopene	Lycopene is present in tomatoes and tomato products and has anti-cancer properties as an antioxidant [177]. (1) Lycopene has been shown to inhibit prostate cancer cell progression and proliferation, induce cell cycle arrest, and promote apoptosis [178]. (2) Lycopene suppresses smoking-induced lung cancer by activating base excision repair [179]. (3) It inhibits gastric cancer growth without impacting epithelial cells [180]. (4) It inhibits liver cancer metastasis by downregulating NADPH oxidase 4 expression [181].	Pre-clinical and clinical trial
Quercetin	Quercetin, a widely occurring phytochemical in common foods, has shown the ability to inhibit a variety of cancer types, including breast, lung, nasopharyngeal, kidney, colorectal, prostate, pancreatic, and ovarian cancers [182,183].	Pre-clinical study and clinical trial
Astaxanthin	Astaxanthin has antioxidant, anti-inflammatory, and anti-apoptotic properties, making it beneficial for preventing or co-treating conditions such as dementia, Alzheimer’s disease, Parkinson’s disease, cardiovascular diseases, and cancer [184].	Pre-clinical study and clinical trial
Tofersen	Tofersen, an antisense oligonucleotide, reduces oxidative stress by lowering the expression of mutant SOD1 protein and has been used in amyotrophic lateral sclerosis (ALS) treatment [185]. However, its role in cancer treatment remains to be explored.	Pre-clinical study and clinical trial

## 6. Challenges and Controversies

### 6.1. The Dual-Edged Function of Antioxidants in Tumor Metabolic Complexity

The use of antioxidants in oncology remains highly controversial. As discussed earlier, antioxidants may shield tumor cells from oxidative stress-induced death, potentially reducing the efficacy of therapies such as chemotherapy and radiotherapy even though they protect normal cells from oxidative stress. Antioxidants such as vitamin C, NAC, and curcumin offer protective effects against oxidative stress, reducing collateral damage during chemotherapy or radiation [162]. Moreover, the lack of consensus on the use of antioxidants is reflected in the variability of study outcomes across different cancer types. For instance, antioxidants such as vitamin E and selenium have been associated with both beneficial effects (e.g., improved quality of life and reduced toxicity from treatments) and harmful effects (e.g., increased risk of cancer progression in some cases) [152]. In addition, tumor cells exhibit metabolic heterogeneity, with some leveraging oxidative stress for survival and proliferation, while others over-activate endogenous antioxidant mechanisms, such as GPX4 or NRF2, to resist external antioxidant treatments. The development of tumor-specific antioxidant delivery systems using nanotechnology to minimize systemic effects and maximize the targeting of tumor cells will be a promising strategy to overcome the heterogeneity. Meanwhile, identifying the metabolic vulnerabilities for specific tumors and personalizing antioxidant therapies are important steps.

### 6.2. Complex Interactions Between Antioxidants and Immune Responses

ROS play a critical role in shaping immune cell function, acting as both signaling molecules and mediators of cytotoxicity. Redox balance is particularly important, as excessive ROS damage cellular components and impair immune function, while insufficient ROS may fail to trigger essential immune responses. Antioxidants modulate innate immunity, particularly by affecting macrophage polarization [186]. ROS levels influence macrophage phenotypes, with high ROS levels favoring the pro-inflammatory M1 phenotype, and low ROS levels promoting the immunosuppressive M2 phenotype [187]. Exogenous antioxidants may thus skew macrophage polarization toward an M2-like state, which supports tumor growth, angiogenesis, and immune evasion. Furthermore, antioxidants impact adaptive immune responses by regulating T cell activation and cytokine secretion. ROS are involved in T cell receptor (TCR) signaling and the transcriptional activation of cytokines such as IFN-γ, IL-2, and TNF-α [87,188]. Excessive antioxidant supplementation may reduce ROS levels below the threshold required for optimal T cell activation, impairing proliferation and cytokine production, and consequently weakening anti-tumor immunity. In addition, naïve T cells and memory T cells predominantly rely on oxidative phosphorylation to meet their energy demands and maintin quiescence [189]. In these cells, mitochondrial ROS serve as key secondary messengers, influencing cell signaling pathways, proliferation, and differentiation [93,190]. However, the exogenous antioxidants may inadvertently disrupt immune cell activity. Antioxidants lower ROS levels, thereby impairing the ability of cytotoxic T lymphocytes (CTLs) and NK cells to kill tumor cells; immune effector cells already face metabolic and functional challenges due to the suppressive and nutrient-deprived conditions.

Moreover, tumor cells may use antioxidants to evade immune surveillance and to avoid ferroptosis [191]. By increasing their antioxidant capacity, tumor cells neutralize the ROS produced by immune cells, such as those released during CD8^+^ T cell- and NK cell-mediated attacks. This adaptation not only enhances tumor cell survival but also exacerbates immune evasion by maintaining a suppressive environment within the TME.

### 6.3. Antioxidant Toxicity and Long-Term Effects

Despite the promising potential of antioxidants in mitigating cancer treatment, long-term supplementation with antioxidants raises several concerns regarding toxicity and potential interference with cancer therapies. Under certain conditions, especially at supraphysiological doses, some antioxidants may paradoxically act as pro-oxidants rather than protectors [192]. For instance, vitamin C can reduce metal ions such as Fe^3+^ to Fe^2+^, which participates in Fenton reactions and produces hydroxyl radicals, thereby increasing oxidative stress. Similarly, polyphenols such as EGCG and quercetin have been shown to exert pro-oxidant activity at high concentrations, resulting in mitochondrial dysfunction and DNA damage. Moreover, long-term use of high-dose antioxidants may interfere with the efficacy of other cancer therapies, including chemotherapy, radiotherapy, and immunotherapy. Chemotherapies, such as platinum-based drugs (cisplatin, carboplatin), rely on ROS generation to induce DNA damage and subsequent cell death in cancer cells. Antioxidants reduce the effectiveness of these drugs by scavenging ROS, thereby mitigating their therapeutic action. Additionally, antioxidants may alter the immune response in cancer patients, potentially dampening the effectiveness of immunotherapies that rely on ROS to activate immune cells and promote tumor destruction. Long-term antioxidant use may also lead to accumulation and toxicity in tissues. For instance, selenium, when taken in high doses, has been associated with selenosis (selenium toxicity), which can manifest in symptoms such as nausea, vomiting, hair loss, and even neurological damage [193].

## 7. Future Directions and Potential Clinical Applications

### 7.1. Optimizing Antioxidant Therapy

The key challenge is balancing the therapeutic benefits of antioxidants—such as reducing oxidative stress and mitigating side effects of treatments such as chemotherapy and radiation—with the potential risks, including the possibility of promoting tumorigenesis or inducing resistance to therapy. Optimizing antioxidant therapy will require a more nuanced approach that considers the specific tumor type, stage of disease, and the molecular context within individual patients.

#### 7.1.1. Personalized Medicine

A promising avenue for optimizing antioxidant therapy involves personalized medicine [194,195]. Cancer patients exhibit considerable heterogeneity in terms of genetic profiles, tumor biology, and responses to treatment. Therefore, antioxidants should be administered based on patient-specific characteristics, which could be determined through genomic and proteomic profiling [196,197]. By identifying genetic markers associated with oxidative stress pathways, clinicians could determine which patients are more likely to benefit from antioxidant supplementation and which may be at risk for negative outcomes. For instance, melanoma and lung cancer may have higher ROS levels as part of their pathophysiology, indicating that antioxidants might be more beneficial in these contexts to limit oxidative damage from chemotherapies [198].

#### 7.1.2. Optimal Timing and Dosage of Antioxidants

Additionally, determining the optimal timing and dosage of antioxidants will be crucial for maximizing their benefits. High-dose antioxidants, such as vitamin C or NAC, have been shown to act as pro-oxidants in certain settings, highlighting the importance of establishing safe dosage ranges [199]. Pharmacokinetic and pharmacodynamic studies to determine the most effective dosing regimens, while minimizing any adverse pro-oxidant effects, need to be optimized. Furthermore, antioxidants could be used as adjuncts to conventional therapies such as chemotherapy and radiotherapy, administered in ways that allow for their potential to protect normal tissues without interfering with the pro-oxidant mechanisms that drive cancer cell death.

### 7.2. Nanoparticle-Based Antioxidants

One of the most promising advancements in antioxidant therapy is the use of nanotechnology to deliver antioxidants specifically to tumor sites, minimizing systemic toxicity while maximizing therapeutic effects [200,201]. Nanoparticles offer several advantages over traditional antioxidant formulations, including targeted delivery, controlled release, and the ability to cross biological barriers that hinder conventional antioxidants, such as the blood–brain barrier [202]. Nanoparticle-based antioxidants can be designed to release their payload in response to specific triggers within the TME, such as elevated ROS levels or acidic pH, thereby providing a more controlled and precise therapeutic effect [203,204]. For example, lipid-based nanoparticles and polymeric nanoparticles have been employed to encapsulate antioxidants such as vitamin C, resveratrol, or glutathione. Natural antioxidant-based nanodrugs have been used to treat atherosclerosis [205], bladder cancer [95], and acute myeloid leukemia [206]. The therapeutic efficacy of these nanoparticles is critically influenced by their design features—including size, surface charge, shape, and surface modification—which affect their circulation time, tumor penetration, and interaction with specific redox pathways. For instance, surface-functionalized nanoparticles can be engineered to selectively accumulate in tumor tissues and release antioxidants in response to redox imbalances, enhancing therapeutic precision and minimizing the disruption of physiological ROS signaling in normal tissues. Furthermore, the ability of nanoparticles to conjugate with other therapeutic agents opens the door for combination therapies, where antioxidants are co-delivered alongside chemotherapeutic drugs or immunotherapies to enhance their efficacy.

### 7.3. Combination Therapies

Combining antioxidants with other therapeutic agents has shown promise in improving clinical outcomes by enhancing the efficacy of chemotherapy, radiotherapy, and immunotherapy [207,208]. Antioxidants modulate oxidative stress, DNA damage responses, and immune functions, offering potential synergistic effects that reduce treatment side effects or boost the activity of other therapies without compromising their effectiveness [209,210]. A key strategy is the use of antioxidants as chemo- and radio-sensitizers. Chemotherapy and radiotherapy often rely on the generation of ROS to induce cancer cell death. Antioxidants protect normal tissues while increasing oxidative stress in tumor cells, making them more susceptible to these treatments. For example, Diosmetin (antioxidant) has been found to enhance cisplatin-based chemotherapy’s efficacy by shielding healthy cells from toxicity while promoting cancer cell death in esophageal squamous cell carcinoma [211].

The integration of antioxidants with immunotherapy represents another promising direction. Immune checkpoint inhibitors, such as PD-1/PD-L1 and CTLA-4 inhibitors, boost the immune system’s ability to target and eliminate cancer cells. The combination of vitamin E with anti-PD-1/PD-L1 antibody therapy improved overall survival rates in patients with various cancers significantly by reinvigorating dendritic cells through targeting SHP1 [212]. However, oxidative stress within the TME hinders immune function and reduces the efficacy of immunotherapies. Antioxidants may improve immune responses by enhancing the activity of immune cells such as T cells, macrophages, and dendritic cells, thereby improving therapeutic outcomes, particularly in patients less responsive to monotherapies.

Antioxidants also show potential in combination with targeted therapies, offering a strategy to combat resistance. For cancers driven by specific oncogenes, such as BRAF or EGFR, antioxidants can reduce oxidative stress-induced pathways that contribute to resistance. For instance, curcumin, a natural antioxidant, has demonstrated enhanced anticancer effects when combined with EGFR inhibitors in preclinical models of non-small cell lung cancer (NSCLC), helping to overcome tumor resistance [213].

## 8. Conclusions and Perspectives

In conclusion, antioxidants hold both great promise and significant challenges in cancer therapy. Their complex dual roles, as both pro-oxidants and antioxidants, necessitate a carefully balanced and personalized approach. The integration of antioxidants with conventional cancer treatments and immunotherapy has the potential to improve clinical outcomes, especially when combined with nanotechnology and biomarker-driven strategies. However, several key issues remain unresolved in the field:

1. What is the optimal dosing regimen for antioxidants in cancer therapy?
2. When should antioxidants be administered to maximize their therapeutic effects in relation to other treatments, such as chemotherapy or radiation?
3. How can we account for patient variability, including genetics and tumor biology, when designing antioxidant-based treatments?
4. What are the most effective methods for measuring antioxidant effects in clinical trials to assess their true impact on tumor biology and treatment outcomes?

Another pressing challenge lies in the complexity and heterogeneity of the tumor microenvironment (TME), which not only shapes cancer progression but also dictates the redox balance within tumors. Antioxidants may either restore redox homeostasis or unintentionally dampen the oxidative stress required for anti-tumor immune responses. Thus, understanding how antioxidants interact with different TME components, such as tumor-associated macrophages, T cells, and stromal cells, is crucial.

Looking forward, antioxidants have the potential to play an increasingly important role in cancer therapy [214]. While the combination of antioxidants with existing therapeutic modalities holds promise, success will depend on resolving these challenges [215]. Refining the therapeutic window, optimizing dosing regimens, and tailoring interventions based on patient-specific molecular profiles are essential for maximizing the therapeutic benefits of antioxidants. Translating preclinical findings into larger-scale clinical trials and integrating systems biology, biomarker development, and novel delivery mechanisms, such as nanoparticles, will play a key role in advancing this field. The future of antioxidants in oncology hinges on the development of personalized, evidence-based treatment regimens that leverage their potential while minimizing risks. As our understanding of cancer biology and antioxidant mechanisms deepens, antioxidants could emerge as a valuable adjunct in cancer care, offering improved treatment outcomes and better overall survival rates for cancer patients.
